# The County Council and Nuisances

**Published:** 1898-06-25

**Authors:** 


### The Hospital

A Journal of

The Medical Sciences and Hospital Administration.

Edited by Sir Henry Burdett, K.C.B., and Solomon C. Smith, M.D., M.R.C.P.

Vol. XXIV.—No. 613.] [June 25, 1898.

### THE COUNTY COUNCIL AND NUISANCES.

It would scarcely be possible to bring to the average Londoner a more welcome piece of intelligence than that the County Council is about to turn away, even if it be but for a brief period, from those regions of high politics in which it has hitherto been the chief pleasure of its least capable members to disport themselves, and to direct its attention to the abatement of nuisances, and generally to rendering urban life more tolerable than most of us have been accustomed to find it. Sir James Paget many years ago described the unchecked prevalence of discordant noises in London as an evil of no slight magnitude in relation to the sick, and as being utterly incompatible with a really high standard of civilisation. An Act of Parliament, passed so long ago as in 1888, empowered the County Council to make by-laws "for the good rule and government of London," and we are told that, ten years having elapsed since the powers were granted, they are now really about to be exercised. The very first object which should be aimed at is the suppression of unnecessary and inharmonious noise. There can be no valid reason why two costermongers should be permitted to proceed slowly along a West End street bawling out their offers of strawberries or other wares in tones which might excite envy in the shade of Stentor himself, and which effectually check conversation in the lower rooms of the adjacent houses. We specify a "West End" street because there is reason to believe that persons of the costermonger class are not themselves painfully affected by shouting, which may even be permissible and convenient in the localities from which they chiefly come. Like children, they often seem to enjoy mere noise for its own sake, and either produce or listen to it with equal pleasure. They have, probably, no wish to inflict discomfort upon others, and no idea that they are doing so. There can be no valid reason why the familiar shriek of "All the weeners," so familiar in the neighbourhood of metropolitan railway stations, should any longer be permitted to split the ears of passers-by; and it might fairly be hoped that its suppression would have some tendency, by the mere withdrawal of suggestion, to check the betting upon horse races now carried on by scores of errand boys and others, who would hardly know what a horse was if they saw it out of an omnibus, and to whom the columns of the so-called "sporting" papers are just so many provocatives to gambling. A still worse evil, from the point of view of respectable residents, is the nocturnal yelling of songs and choruses by parties of half-tipsy hobbadehoys, as they reel homewards when the public-houses are closed. There can be no worse abuse of the liberty of the subject than thus to arouse industrious or ailing people from their first sleep; and the district to the west and north-west of Marylebone, in which the ordinarily "quiet" streets contain many nursing homes and similar establishments, are especially prone to suffer in this manner from the gangs of boys who harbour in the adjacent slums, and who are constant sources of trouble to the police. Many minor or stationary varieties of noise should at the same time receive attention, such, for example, as the steam organs, shooting galleries, and roundabouts, for which comparatively unobjectionable places might no doubt sometimes be found, but which are often pitched where they constitute undoubted nuisances, such as should be easily dealt with by the law. If the "Progressive" majority of the Council will really consent to give us the promised or expected boon of peace and quietness, they will tempt many Londoners to hasten to the polls in their support at the period of the next election. It seems hard to have waited ten years for reform, but "late" is proverbially better than "never," and we must not look the gift-horse too closely in the mouth.

Next to diminished noise, the thing most wanted in London, or at least in some parts of it, for vestries differ widely among themselves, is increased cleanliness. We are not sure whether the Act of 1888, already referred to, would cover questions of scavenging; but, if not, it may be hoped that the expected London Municipal Reform Act may do what is required in this direction. By an odd coincidence, a peculiarly medical quarter of London is the worst off, in this respect, of any with which we are acquainted. The filth of some of the principal residential streets of Marylebone, of Harley Street, and Queen Anne Street, for example, might rival, if it would not surpass, the filth of Pekin. The carriage roads in wet weather are like ploughed fields, and pedestrians carry the mud to the foot pavements, which are never washed down from one year's end to another. Whatever attempts at cleansing are made, are made at the most inconvenient period of the day; and we have seen a dustman's cart at the house of a leading physician at eleven o'clock in the forenoon, and pools of slush, which had been swept by a revolving brush from the centre of the carriage way, occupying a width of four or five feet next to the foot pavement, and rendering it impossible to cross except by leaping or wading. We must not expect too much at once, but even a beginning in the way of the abatement of nuisances will be eagerly welcomed by Londoners, and will certainly excite that lively gratitude which is said to be associated with an expectation of further favours to come.

